# A novel lithium/sulfur battery based on sulfur/graphene nanosheet composite cathode and gel polymer electrolyte

**DOI:** 10.1186/1556-276X-9-137

**Published:** 2014-03-21

**Authors:** Yongguang Zhang, Yan Zhao, Zhumabay Bakenov

**Affiliations:** 1 Nazarbayev University, 53 Kabanbay Batyr Avenue, Astana 010000, Kazakhstan; 2 Institute of Batteries, 53 Kabanbay Batyr Avenue, Astana 010000, Kazakhstan

**Keywords:** Graphene nanosheet, Lithium/sulfur battery, Nanostructured sulfur cathode, Gel polymer electrolyte, Poly(vinylidene fluoride-co-hexafluoropropylene)/poly(methylmethacrylate)/silicon dioxide polymer matrix, 82.47.Aa, 82.45.Gj, 62.23.Kn

## Abstract

A novel sulfur/graphene nanosheet (S/GNS) composite was prepared via a simple ball milling of sulfur with commercial multi-layer graphene nanosheet, followed by a heat treatment. High-resolution transmission and scanning electronic microscopy observations showed the formation of irregularly interlaced nanosheet-like structure consisting of graphene with uniform sulfur coating on its surface. The electrochemical properties of the resulting composite cathode were investigated in a lithium cell with a gel polymer electrolyte (GPE) prepared by trapping 1 mol dm^−3^ solution of lithium bistrifluoromethanesulfonamide in tetraethylene glycol dimethyl ether in a polymer matrix composed of poly(vinylidene fluoride-co-hexafluoropropylene)/poly(methylmethacrylate)/silicon dioxide (PVDF-HFP/PMMA/SiO_2_). The GPE battery delivered reversible discharge capacities of 809 and 413 mAh g^−1^ at the 1st and 50th cycles at 0.2C, respectively, along with a high coulombic efficiency over 50 cycles. This performance enhancement of the cell was attributed to the suppression of the polysulfide shuttle effect by a collective effect of S/GNS composite cathode and GPE, providing a higher sulfur utilization.

## Background

Lithium-ion batteries are leading power sources for portable applications from small consumer electronics to electricity-powered transport. Despite this, their wider application is restricted due to the limited energy density of available cathode materials. Alternative cathode materials with high energy density and low cost are thus needed [1]. Sulfur is very attractive as a cathode material for the next-generation high-energy rechargeable lithium batteries because of its advantages of a large theoretical capacity of 1,672 mAh g^−1^, which is the highest among all known cathode materials, low cost, and environmental friendliness [2-4]. Despite this, due to its insulating nature, large volume changes during electrochemical processes, and the solubility of the polysulfides formed during these processes, the practical application of sulfur as a cathode in lithium rechargeable batteries has not been successful yet [5,6].

Therefore, intensive efforts have been devoted to overcome the mentioned problems. The preparation of sulfur/carbon and sulfur/conductive polymer composites has received considerable attention, and recent results show that the sulfur/carbon composites benefit from their hierarchical design resulting in the performance improvement [7-21]. Microporous and mesoporous carbon nanoparticles [10,11], carbon nanotubes [13], and graphene sheets [14-16] have been employed to encapsulate sulfur. However, the preparation techniques used to obtain these materials have the disadvantages of side products and prolonged and complicated processing, increasing the final product cost [10].

In this work, we report on the preparation of a novel sulfur/graphene nanosheet (S/GNS) composite via a simple ball milling of sulfur and commercial multi-layer graphene nanosheets, followed by a heat treatment, and investigation of its physical and electrochemical properties as a cathode for Li|S batteries.

Diffusion of lithium polysulfides is largely determined by the electrolyte components; adopting an appropriate electrolyte is critical to promote the performance of Li|S batteries [22]. In previous studies [9,10], it was shown that a gel polymer membrane can act as a physical barrier, controlling the cathode reaction product dissolution, restricting their diffusion from the cathode, and thus preventing their reaction at the anode side. Herein, in the present work, to further enhance the battery performance, a common liquid organic electrolyte was replaced with an original gel polymer electrolyte, formed by trapping a liquid electrolyte in tetraethylene glycol dimethyl ether electrolyte in a poly(vinylidene fluoride-co-hexafluoropropylene) (PVDF-HFP)/poly(methylmethacrylate) (PMMA) polymer matrix doped with silicon dioxide (SiO_2_) nanoparticles. The electrochemical and structural properties of this GPE and the electrochemical performance of Li|S/GNS batteries with this GPE were also investigated.

## Methods

**Figure 1:**
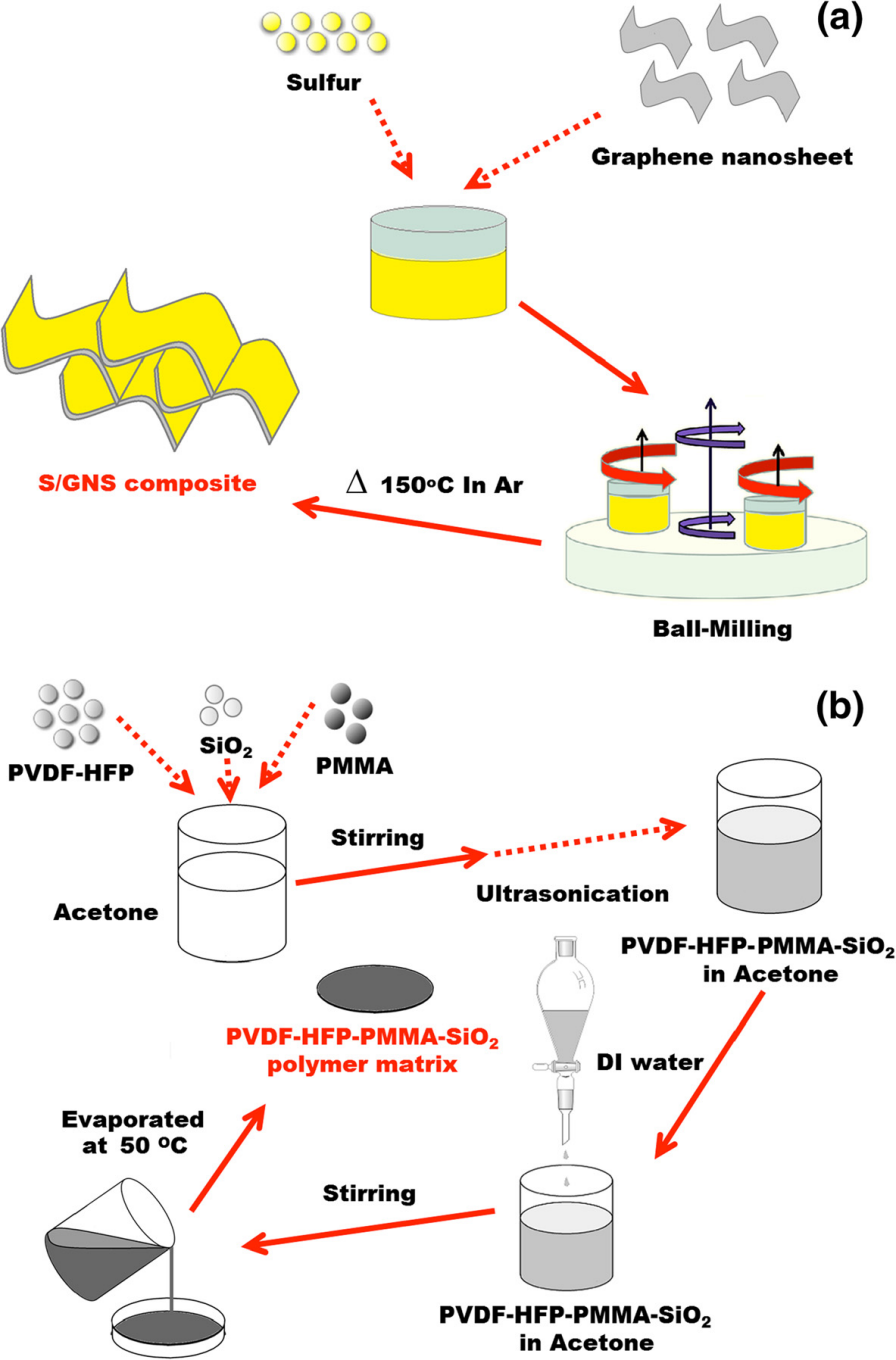
**Schematics of the preparation process.** Schematic diagrams of the synthesis of **(a**) S/GNS composite and **(b)** PVDF-HFP/PMMA/SiO_2_ polymer matrix.

The preparation of S/GNS composite is represented in Figure 1a. Sulfur (high purity, GOST 127.1, Tengizchevroil, Atyrau, Kazakhstan) and graphene nanosheets (US Research Nanomaterials Inc., Houston, TX, USA) were mixed in the weight ratio of 3:1 and wet ball-milled (Pulverisette 7 classic line, Fritsch, Idar-Oberstein, Germany) at 800 rpm for 3 h with ethanol as a dispersant. The precursor mixture was further dried in a vacuum oven at 60°C for 3 h, dry ball-milled at 600 rpm for 6 h, and then heat-treated at 150°C for 6 h in a tube furnace in argon. The sulfur content in the final S/GNS composite was 65 wt% as determined by chemical analysis (CHNS, vario MICRO cube, Elementar, Hanau, Germany).

The preparation of the GPE is schematically represented in Figure 1b. Among other polymer pore-making technologies, we adopted the phase inversion method to obtain a porous structured system through a solvent exchange route [23,24]. The membrane is formed by polymer precipitation, which occurs as a consequence of concentration variations following diffusive interchange between the solvent (acetone) and the non-solvent (water). PVDF-HFP (KynarFlex 2801, Arkema Inc., Philadelphia, PA, USA), PMMA (average molecular weight 350,000 g mol^−1^, Sigma-Aldrich, St. Louis, MO, USA), and SiO_2_ nanopowder (US Research Nanomaterials, Inc.) were added to acetone in a weight ratio of 3:2:0.25 under stirring followed by sonication. Deionized water was then added dropwise and the mixture was continuously stirred for 3 h. The resulting slurry was cast on an aluminum plate and the solvent was evaporated overnight at ambient temperature. The resulting membrane was dried under vacuum at 50°C for 5 h. The resulting mechanically stable membranes, approximately 80 μm thick, were activated inside an argon-filled glove box (As One Co., Osaka, Japan) by immersion in a 1 mol dm^−3^ solution of lithium bistrifluoromethanesulfonamide (LiTFSI) in tetraethylene glycol dimethyl ether (99.95% purity, Sigma-Aldrich). The liquid uptake (%) was determined using the relation (*W*_2_ − *W*_1_) × 100/*W*_1_, where *W*_1_ and *W*_2_ denote the respective weights of the polymer electrolyte before and after absorbing the lithium salt solution [25].

The S/GNS composite surface morphology was examined by field emission scanning electron microscopy (SEM; JSM-6490, JEOL, Akishima, Tokyo, Japan). The interior structure of the composite was observed by transmission electron microscopy (TEM; High Voltage LIBRA 120, Сarl Zeiss, Oberkochen, Germany) with energy-dispersive X-ray spectroscopy (EDX). The ionic conductivity of the GPE was determined at 25°C by electrochemical impedance spectroscopy (EIS) over the frequency range from 0.1 Hz to 1 MHz using potentiostat/galvanostat VMP3 (Biologic, Claix, France), sandwiching the GPE membranes between two blocking stainless steel electrodes. The electrochemical stability window of GPE was determined by cyclic voltammetry (CV) conducted with VMP3 in coin-type cells where GPE was interleaved between lithium metal and stainless steel electrodes.

The electrochemical performance of the S/GNS composite cathode was investigated in coin-type cells (CR2032) with PVDF-HFP/PMMA/SiO_2_ GPE. The cell was composed of a lithium metal anode and the S/GNS composite cathode separated by the GPE film. The cathode is comprised of 80 wt% S/GNS composite, 10 wt% acetylene black (AB; 99.5% purity, MTI, Richmond, CA, USA) as a conductive agent, and 10 wt% polyvinylidene fluoride (PVDF; 99.5% purity, MTI) as a binder. These materials were dispersed in 1-methyl-2-pyrrolidinone (NMP; ≥99% purity, Sigma-Aldrich). The resultant slurry was spread onto aluminum foil using a doctor blade and dried at 50°C for 12 h. The resulting cathode film was used to prepare the cathodes by punching circular disks of 1 cm in diameter. The coin cells were assembled in high-purity argon (99.9995%) atmosphere. The cells were tested galvanostatically on multi-channel battery tester (BT-2000, Arbin Instruments, College Station, TX, USA) between 1 and 3 V vs. Li^+^/Li. The applied currents and specific capacities were calculated on the basis of the weight of S in the cathode.

## Results and discussion

**Figure 2:**
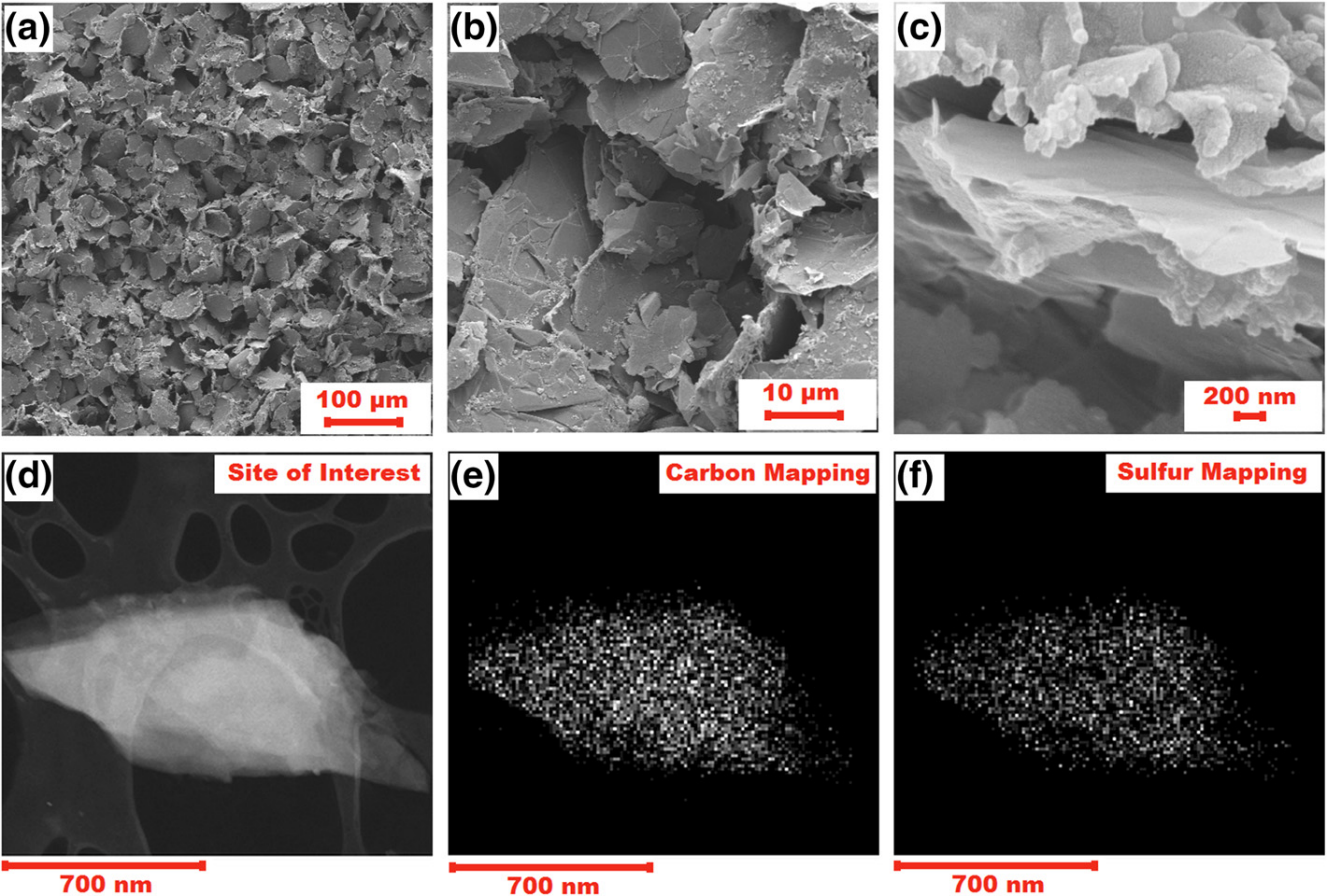
**Morphology of the synthesized S/GNS composite. (a to c)** SEM image of S/GNS composites at different magnifications. **(d to f)** EDX mapping showing distribution of carbon and sulfur.

Figure 2a,b,c exhibits the SEM images of the S/GNS composite at different magnifications. The data of Figure 2a,b show that after the high-speed ball milling the composite contains graphene nanosheets remarkably reduced in size compared with the initial graphene used for the composite synthesis (not shown). At the higher magnification (Figure 2c), it can be clearly seen that GNS sheets are covered with sulfur, and irregular stacks of interlaced nanosheet-like structure were formed. The EDX mapping (Figure 2d,e,f) confirms the homogeneous distribution of the components of the S/GNS composite. It could be suggested that the graphene nanosheets may act as nano-current collectors for the sulfur particles and enhance the conductivity of the composite. On the other hand, the size reduction of graphene and formation of disordered and hollow structure of the composite agglomerates create the pathways for the electrolyte and Li-ion transport providing enhanced activity of the composite. These structural advantages of the composite are favorable for the cathode rate capability, which was further observed in the electrochemical studies.

**Figure 3:**
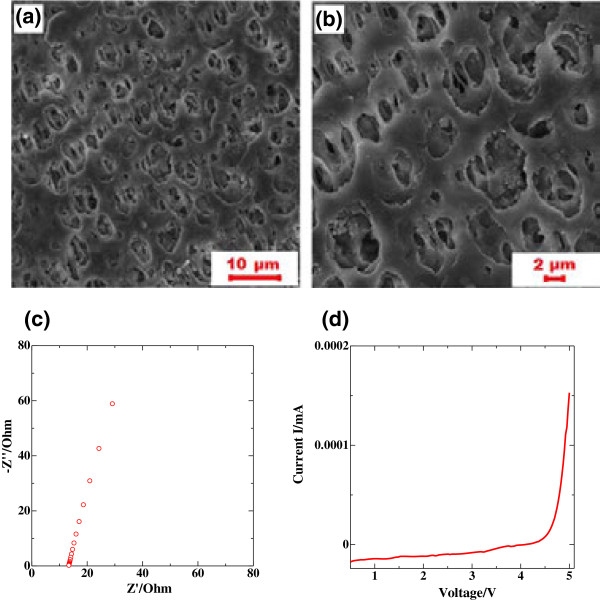
**Morphology, ionic conduction, and electrochemical stability of the synthesized GPE. (a, b)** SEM images of PVDF-HFP/PMMA/SiO_2_ polymer matrix at different magnifications. **(c)** Impedance spectra of as-prepared gel polymer electrolyte. **(d)** CV profile of Li/GPE/SS cell (scan rate 0.1 mV s^−1^).

Figure 3a,b presents the SEM images of the PVDF-HFP/PMMA/SiO_2_ polymer matrix at different magnifications. The membrane is highly porous, and the pore diameters range from 1 to 5 μm. This porous structure grants a high surface area to the membrane and effectively enhances its absorption ability, and the liquid uptake by the membrane was as high as 71 wt%. The obtained GPE was a self-standing transparent film without visible leakage of liquid electrolyte. The ionic conductivity of GPEs strongly depends on the amount of liquid electrolyte embedded in the pores of a polymer membrane, and it is accepted that the absorbed electrolyte solution acts as a medium for ion transport through the polymer matrix [26,27]. A typical EIS plot for the PVDF-HFP/PMMA/SiO_2_ composite sandwiched between two stainless steel blocking electrodes is shown in Figure 3c. No semicircles were observed in the high-frequency part of the Nyquist plot, implying that the polymer electrolyte has a high integrity and its total conductivity mainly results from the ionic conduction [28,29]. The GPE membrane exhibited a high room temperature ionic conductivity of 3.12 mS cm^−1^. The CV data of the GPE (Figure 3d) do not show any breakdown or abrupt current rise during cycling up to 4.5 V vs. Li^+^/Li, confirming that the GPE is electrochemically stable in the operation range of Li|S cell between 1 and 3 V vs. Li^+^/Li.

**Figure 4:**
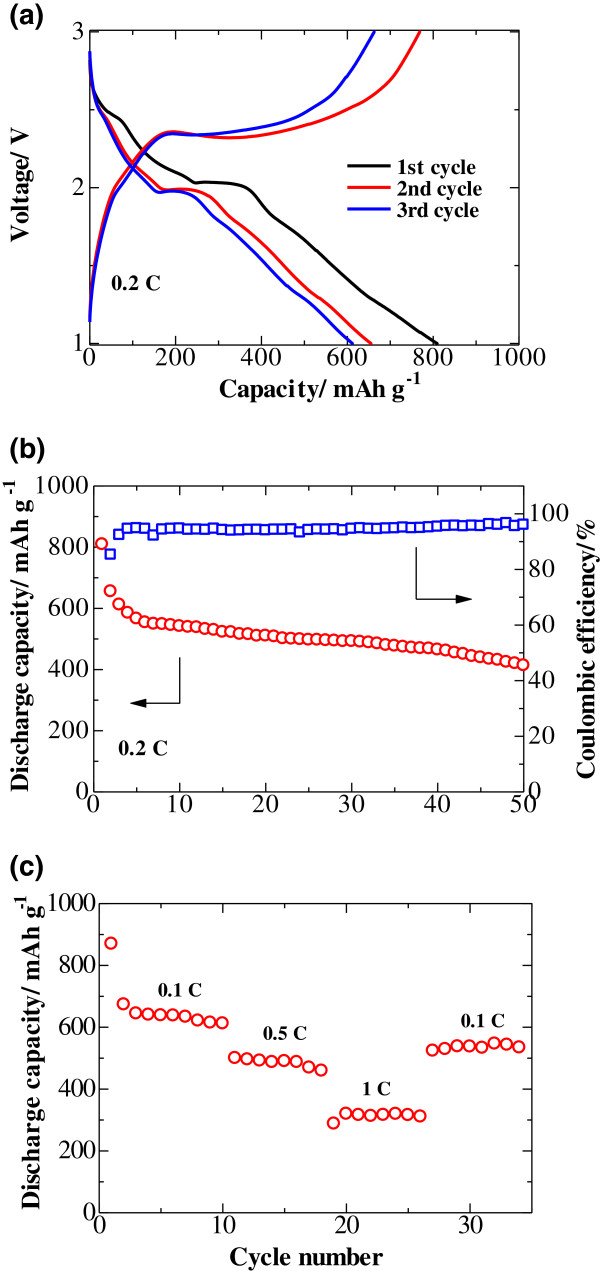
**The electrochemical performance of the Li/GPE/S cell with the S/GNS composite. (a)** The initial discharge/charge profiles and **(b)** cycle performance of the gel polymer cell with S/GNS composite cathode at 0.2C. **(c)** Rate capability performance of the gel polymer cell with S/GNS composite cathode.

The electrochemical performance of the Li|GPE|S cell with the S/GNS composite is presented in Figure 4. The galvanostatic charge–discharge profiles and cycling performance of the cells are depicted in Figure 4a,b. The discharge curves (Figure 4a) show two plateaus that can be assigned to the two-step reaction of sulfur with lithium [9,10]. The first plateau at about 2.4 V is related to the formation of higher-order lithium polysulfides (Li_2_S_*n*_, *n* ≥ 4), which are soluble in liquid electrolyte. The following electrochemical transition of these polysulfides into lithium sulfide Li_2_S_2_/Li_2_S is associated to a prolonged plateau around 2.0 V. The kinetics of the latter reaction is slower than that of the polysulfide formation, which is reflected by the length of the plateaus [6]. Figure 4b presents the cycling performance of the Li|GPE|S cell with the S/GNS composite cathode. The cell delivers a high initial discharge capacity of about 809 mAh g^−1^ at 0.2C rate and exhibits an enhanced cyclability. This indicates that a combination of the S/GNS composite cathode and PVDF-HFP/PMMA/SiO_2_ GPE plays a significant role of retarding diffusion of the polysulfides out of the cathode area and suppressing their transport towards the anode side (shuttle effect). The coulombic efficiency data presented in the same figure confirm this suggestion and reach 95%. For further clarification of the effects of S/GNS composite and GPE on the cell performance, its rate capability performance was investigated. As illustrated in Figure 4c, the cell delivers a reversible discharge capacity of 638 mAh g^−1^ at 0.1C. Although the reversible capacity gradually decreases with the increase in current density, the system still delivers specific discharge capacity of 316 mAh g^−1^ even at 1C, i.e., the high-rate operation is affordable by the system due to a good ionic conductivity of the GPE and an enhanced conductivity of the graphene containing sulfur composite cathode. Upon the following reduction of the C-rate to 0.1C, the cell recovers about 85% of its initial reversible capacity (538 mAh g^−1^). This suggests that both the homogeneous dispersion of nanoscopic sulfur in the layers covering the highly conductive GNS nanosheets, which act as nano-current collectors, provide remarkably enhanced lithium-ion transportation.

## Conclusions

A novel S/GNS composite with irregular interlaced nanosheet-like structure and homogeneous distribution of the components was successfully prepared via a simple ball milling of sulfur with commercial multi-layer graphene nanosheets, followed by a heat treatment. This composite was studied in a lithium cell with an original gel polymer electrolyte, 1 mol dm^−3^ of LiTFSI in PVDF-HFP/PMMA/SiO_2_ polymer electrolyte, prepared by phase separation. The GPE exhibited a pore-rich structure, a high ability to absorb liquid electrolyte exceeding 71 wt%, and a high ionic conductivity at ambient temperature. The Li|GPE|S cells exhibited a high initial specific discharge capacity and maintained a reversible discharge capacity of 413 mAh g^−1^ after 50 cycles at 0.2C, along with a high coulombic efficiency. Due to a combined positive effect of the nanosheet-like structure of conductive S/GPE composite cathode, retaining the S cathode reaction products-polysulfides, and a highly conductive GPE as a physical barrier for these products’ shuttle, the system could deliver reversible capacity of 316 mAh g^−1^ even at 1C. The results of this work show that the S/GNS composite cathode prepared in this work via a simple preparation technique, in combination with the original GPE, provides a promising way to develop the Li|S battery with very attractive overall performances and, due to its simplicity, could be a good choice for the scale-up technology for Li/S batteries.

## Abbreviations

AB: acetylene black; CV: cyclic voltammetry; EDX: energy-dispersive X-ray spectroscopy; EIS: electrochemical impedance spectroscopy; GPE: gel polymer electrolyte; LiTFSI: lithium bistrifluoromethanesulfonamide; NMP: 1-methyl-2-pyrrolidinone; PVDF: polyvinylidene fluoride; PVDF-HFP/PMMA/SiO2: poly(vinylidene fluoride-co-hexafluoropropylene)/poly(methylmethacrylate)/silicon dioxide; S/GNS: sulfur/graphene nanosheet; SEM: scanning electron microscopy; TEM: transmission electron microscopy.

## Competing interests

The authors declare that they have no competing interests.

## Authors’ contributions

YGZ and ZB conceived and designed the experiments and wrote the manuscript. YGZ and YZ performed the experiments. YGZ, YZ, and ZB analyzed the data. ZB contributed reagents/materials/analysis tools. All authors read and approved the final manuscript.
